# Epigenetics in Canine Mammary Tumors: Upregulation of miR-18a and miR-18b Oncogenes Is Associated with Decreased *ERS1* Target mRNA Expression and ERα Immunoexpression in Highly Proliferating Carcinomas

**DOI:** 10.3390/ani13061086

**Published:** 2023-03-17

**Authors:** Jessica Maria Abbate, Francesca Arfuso, Kristian Riolo, Fabiano Capparucci, Barbara Brunetti, Giovanni Lanteri

**Affiliations:** 1 Department of Veterinary Sciences, University of Messina, Polo Universitario Annunziata, 98168 Messina, Italy; jabbate@unime.it (J.M.A.); farfuso@unime.it (F.A.); 2 Department of Chemical, Biological, Pharmaceutical and Environmental Sciences, Polo Universitario Papardo, 98166 Messina, Italy; fcapparucci@unime.it (F.C.); glanteri@unime.it (G.L.); 3 Department of Veterinary Medical Sciences, University of Bologna, 40064 Bologna, Italy; b.brunetti@unibo.it

**Keywords:** canine mammary tumors, epigenetics, ERα, *ESR1*, miR-18a, miR-18b, miRNAs, oncomiR, Ki67 index

## Abstract

**Simple Summary:**

Canine mammary neoplasms commonly lose ERα expression along with an increase in stage and grade, negatively correlating with patient prognosis. The *ESR1* gene encodes ERα and represents an important target mRNA for miR-18a and miR-18b, described as overexpressed oncogenes in canine mammary carcinomas. In this study, we demonstrate a negative correlation between the expression of miR-18a and miR-18b and their target *ESR1* gene. Notably, a significant overexpression of oncogenic miRNAs was observed in malignant canine mammary tumors (CMTs) compared with the non-neoplastic mammary gland and benign CMTs. In contrast, the expression of target *ESR1* mRNA was significantly downregulated along with the increase in tumor grade, and was associated with the progressive loss of ERα immunoexpression. It is noteworthy that the overexpression of miR-18a and miR-18b was observed in malignant tumors with increased proliferation of tumor cells. The results suggest a central role of miR-18a and miR-18b in the pathophysiology of canine mammary tumors, also representing promising biomarkers with predictive and prognostic value.

**Abstract:**

The expression of miRNAs is one of the main epigenetic mechanisms responsible for the regulation of gene expression in mammals, and in cancer, miRNAs participate by regulating the expression of protein-coding cancer-associated genes. In canine mammary tumors (CMTs), the *ESR1* gene encodes for ERα, and represents a major target gene for miR-18a and miR-18b, previously found to be overexpressed in mammary carcinomas. A loss in ERα expression in CMTs is commonly associated with poor prognosis, and it is noteworthy that the downregulation of the *ESR1* would appear to be more epigenetic than genetic in nature. In this study, the expression of *ESR1* mRNA in formalin-fixed, paraffin-embedded (FFPE) canine mammary tumors (CMTs) was evaluated and compared with the expression levels of miR18a and miR18b, both assessed via RT-qPCR. Furthermore, the possible correlation between the miRNA expression data and the immunohistochemical prognostic factors (ERα immunoexpression; Ki67 proliferative index) was explored. A total of twenty-six FFPE mammary samples were used, including 22 CMTs (7 benign; 15 malignant) and four control samples (three normal mammary glands and one case of lobular hyperplasia). The obtained results demonstrate that miR-18a and miR-18b are upregulated in malignant CMTs, negatively correlating with the expression of target *ESR1* mRNA. Of note, the upregulation of miRNAs strictly reflects the progressive loss of ERα immunoexpression and increased tumor cell proliferation as measured using the Ki67 index. The results suggest a central role of miR-18a and miR-18b in the pathophysiology of canine mammary tumors as potential epigenetic mechanisms involved in ERα downregulation. Moreover, as miRNA expression reflects ERα protein status and a high proliferative index, miR-18a and miR-18b may represent promising biomarkers with prognostic value. More detailed investigations on a larger number of cases are needed to better understand the influence of these miRNAs in canine mammary tumors.

## 1. Introduction

Epigenetic mechanisms are critical for the physiological maintenance of tissue-specific gene expression patterns in mammals [1,2]. Changes in the epigenetic background lead to altered gene expression and function and thereby cellular phenotype, and are considered to be “hallmarks” of cancer [1]. In particular, the expression of cancer-associated genes can be silenced, decreased or increased with no structural changes in the DNA sequence [1]. It is noteworthy that the reversible nature of epigenetic changes and their central role in cancer and malignant cellular transformation, has led to the emergence of the promising field of epigenetic therapy for treatment of cancer [2]. 

The expression of non-coding RNAs, especially small (17–24 nucleotides) non-transcribed RNA molecules (microRNAs; miRNAs; miRs), represents one of the key epigenetic mechanisms responsible for the regulation of gene expression in mammals [3,4]. miRNAs are tissue-specifically expressed and are capable of orchestrating a broad range of biological processes and molecular functions, post-transcriptionally regulating gene expression and protein synthesis through the sequence-specific inhibition of messenger RNA (mRNA) [4,5,6]. Additionally, besides the role of gene silencing, miRNAs are also able to increase the expression of target genes through a process known as RNA activation [5,6,7]. In many human and animal cancers, the amplification and deletion of miRNA loci have been detected, and the altered expression of miRNAs can be achieved through different mechanisms, including chromosomal abnormalities and epigenetic modifications [8]. In particular, tumor development can be encouraged by the overexpression of specific miRNAs that silence tumor suppressor genes or through the silencing of specific miRNAs that target oncogenes [9]. It is noteworthy that among several epigenetic mechanisms, miRNAs have been confirmed to play a crucial role in the pathophysiology of breast cancer and several basic and interventional attempts to target epigenomic modifiers in breast cancer have recently been shown to be successful [10,11]. Furthermore, due to their relevance in many cellular pathways, miRNA dysregulation is the most studied epigenetic alteration in canine mammary cancer [12]. 

Mammary tumors are commonly diagnosed in intact bitches and the reproductive status and endocrine microenvironment would seem to play central roles in the pathogenetic mechanisms [13,14,15]. In particular, sexual hormones such as estradiol (E2) have an important role in mammary carcinogenesis, promoting the latter tumor initiation and progression through its binding to the growth-promoting estrogen receptor a (ERα) [14,16,17]. ERα functions as a ligand-activated transcription factor and works by promoting the transcription of pro-proliferative genes, also suppressing pro-apoptotic genes in cells and thereby encouraging cell proliferation and predisposing it to genetic mutations [14,16,17,18]. Most canine patients with mammary tumors express high tissue levels of ERα as well as high serum E2 levels (>35 pg/mL) [19,20], although a negative correlation between ERα expression and histological differentiation is observed, as malignant high-grade canine mammary tumors (CMTs) tend to be ERα-negative [21,22]. Of note, ERα expression would allow for the prediction of the response to antiestrogen hormone therapy, due to its relevance as a therapeutic target in breast cancer, and malignancy with ER expression may have a better prognosis while low ERα expression has been associated with increased tumor growth and/or a poorer prognosis in both human and canine patients [16,21,23,24,25,26].

The *ESR1* gene encodes for ERα and the histological protein expression closely reflects the gene expression pattern [20]; in most aggressive breast cancers, a loss of the ERα has been attributed to methylation of the CpG island in the *ESR1* gene promoter [27]. Conversely, no differences were observed in the proportion of the CpG island methylation in the canine *ESR1* gene when comparing normal mammary glands to malignant mammary tumors [27]. Therefore, unlike in breast cancer, lower ERα expression in malignant CMTs does not appear to be induced by DNA methylation and would be induced by as yet unknown causes [27]. It is noteworthy that another mechanism affecting ER expression in breast cancer is a change in miRNA expression profiles [28,29]. Indeed, more than 50% of miRNAs reside in cancer-associated genes, and their role as regulatory molecules in breast cancer pathophysiology has been established through interaction with ERα [29]. Non-coding RNAs participate in carcinogenesis by regulating the expression of protein-coding cancer-associated genes, and it has been shown that mammalian miRNAs predominantly reduce target mRNA expression with reduced protein production [3]. Therefore, changes in mRNA levels strictly reflect the impact of miRNAs on gene expression and suggest that the destabilization of target mRNA is the predominant reason for reduced protein production [3]. 

Recently, we have demonstrated that oncogenes miR-18a and miR-18b are overexpressed in malignant CMTs compared with normal mammary tissues, and it is noteworthy that both miRNAs are predicted to target *ESR1* mRNA with a very high target score (miRDB target score of 99) [30]. In agreement, both miR-18a and miR-18b have been found to be upregulated in canine-mammary-cancer-derived cell lines [31], and in the sera of dogs with metastatic mammary carcinomas [32], and also, they have been predicted to target the *ESR1* gene. However, to the best of the authors’ knowledge, no study has previously investigated the expression of miRNAs and *ESR1* target mRNA in canine mammary tumors. Therefore, in this study we aimed to investigate the expression of *ESR1* mRNA via real-time quantitative polymerase chain reaction (RT-qPCR) in formalin-fixed, paraffin-embedded (FFPE) CMTs and to compare the expression data with those of miR-18a and miR-18b, as previously assessed [30]. Furthermore, in order to evaluate the prognostic value of these miRNAs, this study aimed to correlate for the first time miRNA gene expression with ERα immunoexpression and the Ki67 proliferative index, as major immunohistochemical prognostic factors. 

## 2. Materials and Methods

### 2.1. Mammary Samples and Histopathology 

Canine mammary tumors were selected among samples referred for diagnostic purposes in the archive of the AniCura Veterinary Hospital “I Portoni Rossi” (Bologna, Italy). Normal mammary gland tissues were obtained from female dogs that underwent necropsy and were used as positive and negative controls for immunohistochemical and molecular investigations. All samples were fixed in 10% buffered formalin and routinely embedded in paraffin blocks. 

Histological examination was performed on three μm thick tissue sections stained with hematoxylin-eosin (HE) by a board-certified pathologist (B.B.), and tumors were classified according to a classification recently published by the Davis-Thompson DVM Foundation [33]. Histological grading for malignant mammary tumors was performed according to Peña et al. [34]. 

### 2.2. Immunohistochemistry

Immunohistochemistry (IHC) was performed on 3 μm thick, paraffin-embedded tissue sections, dewaxed in xylene and rehydrated in ethanol, using the following antibodies: mouse monoclonal estrogen receptor alpha (ERα) (clone C311; Santa Cruz Biotechnology, Santa Cruz, CA, USA; dilution 1:40) and monoclonal mouse Ki67 (clone MIB-1, Dako, Denmark; dilution 1:600). The inhibition of endogenous peroxidases was performed using 3% H_2_O_2_ in methanol for 30′ and antigens were revealed using citrate buffer pH 6.0 and heating in a microwave at 750W for two 5 min cycles and four 5 min cycles for ERα and Ki67, respectively. Tissue sections were pre-incubated for 30 min at room temperature with blocking solution and incubated at 4 °C overnight with primary antibodies. The binding sites were revealed by a secondary antibody, diluted 1:200 in blocking solution (goat anti-mouse polyclonal; Dako, Denmark) and amplified using a commercially available avidin-biotin peroxidase kit (VECTASTAIN ABC Kits); 3,3′-Diaminobenzidine (DAB) was used as the chromogen (ACH500-IFU, ScyTek Laboratories). Slides were counterstained using Meyer’s hematoxylin. Negative controls were included in each IHC reaction, omitting the primary antibodies. Positive internal controls were included and consisted of normal canine mammary glands and uteruses for ERα, and normal canine intestines for Ki67.

The immunoexpression of ERα was evaluated semi-quantitatively according to the Allred scoring system [35]. Briefly, the Allred score (total score, TS) is the sum of the percentage of stained cells (PS) and the intensity of immunolabeling (IS). The total score ranged from 0 or 2 to 8 (Table 1), and the cut-off for considering ER-positive tumors was TS ≥ 3 [24,36]. 

The Ki-67 index was expressed as a percentage by counting the number of positive nuclei in 500 neoplastic cells. A cut-off of 33.3% of Ki67-postive neoplastic nuclei was used to distinguish between high-proliferative vs. low-proliferative tumors [37]. Manual image analysis (Image J software, National Institute of Health, Bethesda, MD, USA) was used to count the immunolabeled cells. 

### 2.3. Molecular Investigations

#### 2.3.1. Total RNA Extraction from FFPE Mammary Samples 

A representative area of tumors and normal/hyperplastic mammary glands was selected under light microscopy, labeled on histological slides and identified in the paraffin block. Two sections 10–15 μm thick of the selected area were sampled, placed in a sterile 1.5 mL tube and used to extract total RNA. Purification of RNA from FFPE tissues was performed using miRNeasy^®^ FFPE Kit (QIAGEN, Milan, Italy; cat. no. 217504) and deparaffinization solution (QIAGEN, Milan, Italy; cat. no. 19093), according to the manufacturer’s instructions. RNA concentration was measured using a nanodrop spectrophotometer (NanoPhotometer N50, Implen, Westlake Village, CA, USA). 

#### 2.3.2. cDNA Synthesis and Real-Time Quantitative PCR (RT-qPCR) for miRNAs

The reverse transcription (RT) reaction and RT-qPCR for miR-18a and miR-18b have been described in detail in our previous publication [30]. Briefly, RT was performed using the miRCURY^®^ LNA RT Kit (QIAGEN, Milan, Italy; cat. no. 339340). Each reaction was set up in a 10-μL final reaction volume using 2 μL of 5× miRCURY RT Reaction Buffer, 4.5 μL of RNase-free water, 1 μL of 10× miRCURY RT Enzyme Mix and 0.5 μL of UniSp6 RNA spike-in, provided as the internal quality control of cDNA synthesis. Finally, 2 μL containing 20 ng of the isolated RNA was added to each RT reaction tube. Reaction tubes were incubated at 42 °C for 60 min and at 95 °C for 5 min. 

The following miRCURY^®^ LNA miRNA PCR Assays (QIAGEN, Milan, Italy; cat. no. 339306) were used: gga-miR-18b-5p (assay ID—YP02100265) and gga-miR-18a-5p (assay ID—YP02100185). Assays hsa-miR-16-5p (assay ID—YP00205702) and hsa-let-7a-5p (assay ID—YP00205727) were used as the reference controls for normalization [31,38]. RT-qPCR was performed in duplicate for each sample in a final reaction volume of 20 μL, containing 10 μL of 2× miRCURY^®^ LNA SYBR Green PCR Kit (QIAGEN, cat. no. 339345–339346), 2 μL of specific miRNA assay, 2 μL of RNase-free water and 6 μL of cDNA (60× diluted), and performed using a Rotor-Gene^®^ Real-Time PCR system (QIAGEN). The cycling conditions were as follows: 95 °C for 2 min, 40 cycles of 95 °C for 10 s and 56 °C for 60 s. The miRNA expression levels were presented in terms of fold change normalized to endogenous controls using Formula 2 ΔΔCq.

#### 2.3.3. cDNA Synthesis and RT-qPCR for ESR1 mRNA

For *ERS1* gene expression, the RT reaction was performed using the Sensiscript^®^ Reverse Transcription Kit (QIAGEN, Milan, Italy; cat. no. 205211) according to the manufacturer’s instructions. Ribonuclease Inhibitor (R1158-2.5KU; Sigma-Aldrich, Co., St. Louis, MO, USA) and oligo-dT primer (Oligo (dT)8; catalog no. BIO-38029; Meridian Bioscience; Germany) was added as it was not supplied in the RT kit. Before setting up the reaction, RNA was denatured for 5 min at 65 °C. RT reaction was set up in a 20-μL final reaction volume, using 2 μL of 10× RT Reaction Buffer, 2 μL of dNTP Mix, 1 μL of Sensiscript Reverse Transcriptase, 1 μL of Oligo-dT primer (10 μM) and 0.5 μL of RNase inhibitor (10 units/μL). Finally, 30 ng of RNA diluted in a variable volume of RNase-free water was added to each tube. RT reactions were incubated at 37 °C for 60 min and obtained cDNA was immediately stored at −20 °C. 

RT-qPCR for the *ESR1* gene was performed using the GoTaq^®^ qPCR Master Mix (Promega, Milan, Italy; cat. no. A6002). Housekeeping genes used as the reference controls for normalization included ribosomal protein S19 (*RPS19*) and hypoxanthine phosphoribosyltransferase 1 (*HPRT*) [39,40]. The sequences of the primers used are given in Table 2. 

RT-qPCR reaction was performed in duplicate for each sample in a 20 μL total reaction volume, containing 10 μL of GoTaq^®^ qPCR Master Mix, 1.5 μL of forward primer (10 μM/μL), 1.5 μL of reverse primer (10 μM/μL) and 5 μL of nuclease-free water. Finally, 2 μL of cDNA template (1:10 diluted) was added. Reactions were performed using a Rotor-Gene^®^ Real-Time PCR system (QIAGEN), with the following cycling conditions: 95 °C for 2 min, 40 cycles of 95 °C for 15 s and 60 °C for 1 min. Gene expression levels are presented in terms of fold change normalized to endogenous controls using Formula 2 ΔΔCq.

### 2.4. Statistical Analysis

The Kruskal–Wallis test was performed to assess statistically significant differences in *ESR1* gene expression values between mammary tissue samples, followed by Dunn’s multiple comparison test. The Mann–Whitney test was applied to evaluate significant differences in Ki67 index between benign (Group B) and malignant CMTs with different grades of malignancy (Group M1–3). Furthermore, the Mann–Whitney test was also applied to evaluate the differences in miR-18a and miR-18b expression levels between tumors with ERα score 4–6 and ERα score 7–8, and between CMTs with a Ki67 index > 33.3% (high proliferative tumors) and a Ki67 index < 33.3% (low proliferative tumors). Pearson’s coefficients were calculated to evaluate a possible correlation between the *ESR1* mRNA and miR-18a and miR-18b expression in benign tumors (Group B) and malignant tumors (Group M1–3). Moreover, the possible correlation between *ESR1* gene expression, miR-18a and miR-18b levels and Ki67 index in benign tumors (Group B) and in malignant tumors (Group M1–3) was investigated. Pearson’s test was applied to evaluate whether miR-18a and miR-18b gene expression correlated with immunohistochemical prognostic factors (i.e., ERα—score; Ki67 index). A linear regression model (y = a + bx) was applied to determine the degree of correlation. A *p* value < 0.05 was considered to be statistically significant.

The statistical analysis was performed using Prism Software v. 9.00 (GraphPad Software Ldt., San Diego, CA, USA, 2020).

## 3. Results

### 3.1. Samples and Immunohistochemistry

Twenty-six mammary samples were used in this study, including twenty-two CMTs (*n* = seven benign; Group B) (*n* = fifteen malignant; Group M1–3), three normal mammary gland specimens and one sample of lobular hyperplasia used as the negative controls (Group C) for immunohistochemical and molecular investigations. Histological grading classified malignant CMTs as follows: Grade I, eight samples (Group M1); Grade II, two samples (Group M2); Grade III, five samples (Group M3). The histological classification of CMTs, the Allred scores for ERα immunoexpression and Ki-67 indexes are shown in Table 3.

**Figure 1:**
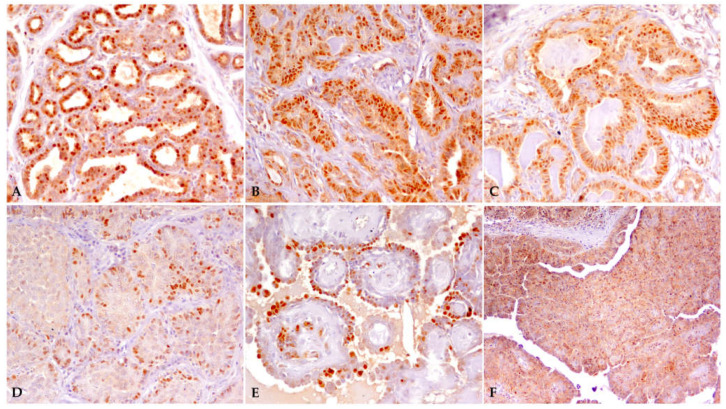
ERα immunoexpression in canine mammary samples. (**A**) Normal mammary gland, TS = 8; (**B**,**C**) tubulopapillary adenoma, TS = 8; (**D**) solid carcinoma, grade III, TS = 3; (**E**) intraductal papillary adenoma, TS = 4; (**F**) solid carcinoma, grade II, TS = 4.

Twenty of the twenty-two CMTs were ERα-positive, whereas two malignant tumors (i.e., intraductal papillary carcinoma, grade I; solid carcinoma, grade III) with TS ≤ 3 were considered to be ERα-negative. ERα immunoexpression of some canine mammary samples with different TS scores is illustrated in Figure 1.

Ki67 positivity was evaluated in all mammary samples. Ki67 positivity across all CMTs was 40.09 ± 22.97% (mean ± SD). Ki67 positivity was 21.97 ± 10.21% and 47.34 ± 22.79% in benign and malignant canine mammary tumors, respectively. No significant differences (*p* > 0.05) were found in the mean Ki67 index between benign tumors (Group B: coefficient of variation, 76.57%; median, 19.60; minimum, 12.00; maximum, 77.20) and malignant CMTs (Group M1–3: coefficient of variation, 48.15%; median, 54.00; minimum, 7.00; maximum, 87.60).

### 3.2. miR-18a and miR-18b gene Expression Levels

**Figure 2:**
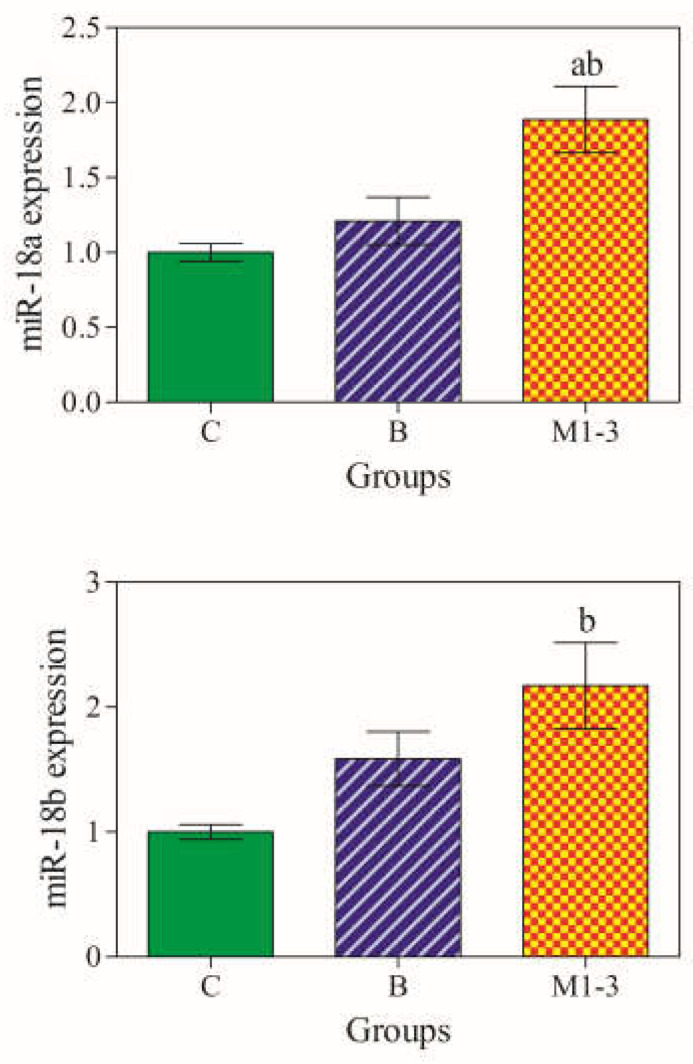
Expression levels of miRNAs in mammary samples. miR-18a was overexpressed in malignant tumors (M1–3; orange color) compared with the control group (C; green color) and benign tumors (B; blue color), whereas miR-18b was overexpressed in malignant tumors (M1–3) compared with the benign tumors (B). Significances (*p* < 0.05) ^a^ vs. Group C and ^b^ vs. Group B.

miRNAs were amplified in all mammary samples and a statistically significant difference in miR-18a and miR-18b expression levels was observed among groups (*p* < 0.05). Notably, mir-18a and miR-18b were significantly overexpressed in malignant tumors (Group M1–3) compared with the normal/hyperplastic mammary gland (Group C). Furthermore, miR-18a expression was also upregulated in malignant CMTs compared with benign tumors (Group B) (Figure 2) [30].

### 3.3. Correlation between miRNAs and Immunohistochemical Prognostic Factors

Canine mammary tumors were divided according to ERα-score (4–6 vs. 7–8) and Ki67 index (low vs. high proliferative tumors; cut-off 33.3%) to compare the mean values of miRNA expression and immunohistochemical prognostic factors (Table 4).

**Figure 3:**
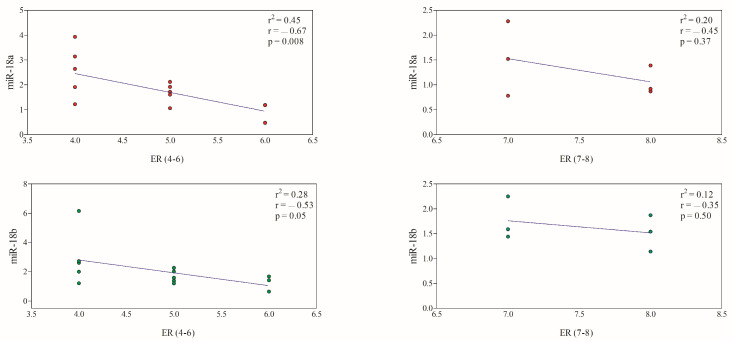
Correlation between miRNA expression and ERα score. A significant negative correlation was observed for both miR-18a (red circles) and miR-18b (green circles) in CMTs with lower ERα immunoexpression. The colored circles (red; green) refer to miRNA expression levels in CMTs.

No significant differences (*p* > 0.05) were found in miR-18a and miR-18b expression levels between CMTs with an Erα score of 4–6 and an ERα score of 7–8, as well as between tumors with a Ki67 index > 33.3% and <33.3%. The expression of miR-18a and miR-18b showed a significant negative correlation with an ERα score of 4–6, while it did not show a significant correlation with the Erα score of 7–8 (Figure 3).

**Figure 4:**
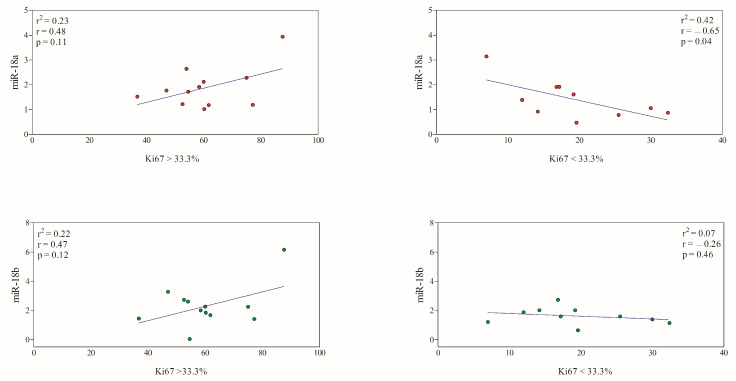
Correlation between miRNA expression and Ki67 index. A significant negative correlation was observed only between miR-18a (red circles) expression levels and Ki67 index < 33.3% (*p* < 0.05). The miR-18b (green circles) gene expression did not show a significant correlation with Ki67 index. The colored circles (red; green) refer to miRNA expression levels in CMTs.

The expression levels of miR-18a showed a significant negative correlation with a Ki67 index < 33.3%, while it was not correlated with a Ki67 index > 33.3%. The miR-18b gene expression did not show a significant correlation with a Ki67 index < 33.3% and >33.3% (Figure 4).

**Figure 5:**
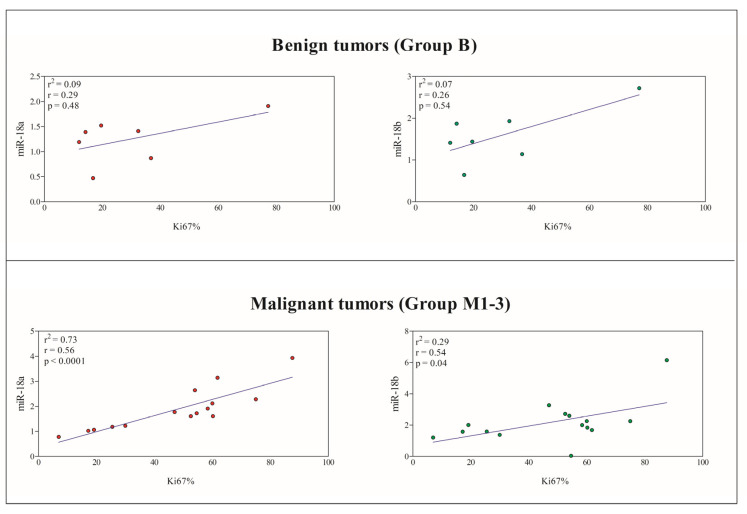
Linear regression obtained between the Ki67 index and the expression of miR-18a (red circles) and miR-18b (green circles) in benign tumors (Group B) and malignant tumors (Group M1–3). A significant positive correlation was found between the Ki67 index and the miR-18a and miR-18b expression levels in malignant CMTs (*p* < 0.05).

Considering benign vs. malignant CMTs, the Ki67 positivity was 21.97 ± 10.21% and 47.34 ± 22.79%, respectively. In this case, a significant positive correlation was found between the Ki67 index and the miR-18a and miR-18b expression levels in malignant tumors (Group M1–3), whereas the Ki67 index of benign tumors was not correlated with miR-18a and miR-18b gene expression (Figure 5). These findings were confirmed by the results of the linear regression model.

### 3.4. ESR1 Gene Expression

**Figure 6:**
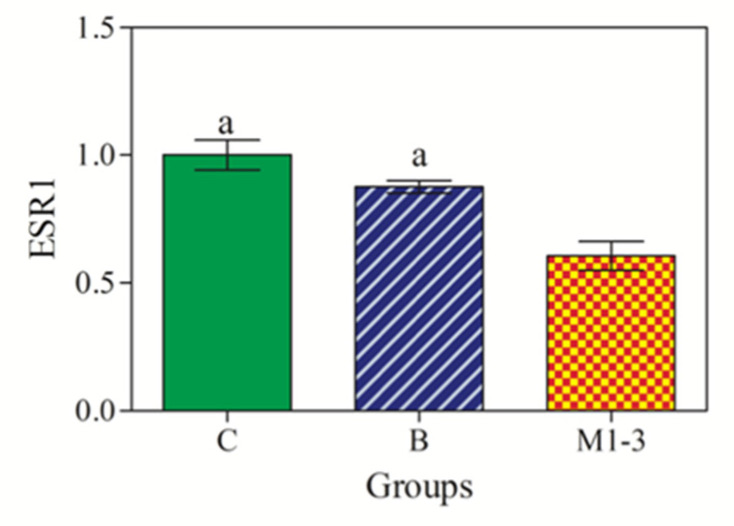
RT-qPCR expression levels of *ESR1* mRNA. *ESR1* expression was downregulated in malignant tumors (M1–3; orange color) compared with control (C; green color) and benign tumors (B; blue color). Significances (*p* < 0.001): a vs. Group M1–3.

As shown in Figure 6, a statistically significant effect of group in *ESR1* mRNA expression was observed, with significant lower *ESR1* gene expression in malignant tumors (Group M1–3) compared with normal/hyperplastic mammary tissues (Groups C) and benign CMTs (Group B) (*p* < 0.001).

According to Pearson’s test results, no significant correlation was found between the *ESR1* mRNA expression levels and miR-18a, miR-18b and Ki67 indexes in both benign and malignant CMTs.

## 4. Discussion

Cancer is a disease with both genetic and epigenetic components, and it is noteworthy that in breast cancer epigenetic dysregulation has a central role through genomic instability [10]. In particular, non-coding RNAs participate in carcinogenesis by regulating the expression of protein-coding cancer-associated genes, and it is noteworthy that among the different molecular mechanisms studied on ER downregulation, the progressive loss of ER expression in breast cancer would appear to be more epigenetic than genetic in nature [41]. Conversely, studies approaching epigenetic mechanisms in canine mammary cancer are still scarce and only few studies investigating miRNA expression have been published, to date [12,31,32].

This study assessed *ESR1* mRNA expression via RT-qPCR in FFPE canine mammary samples to compare data with previously investigated miR-18a and miR-18b predicted to target ERS1 mRNA, as a possible epigenetic mechanism responsible for the loss of the estrogen receptor alpha in canine mammary cancer. Furthermore, miR-18a and miR-18b expression was also correlated with major immunohistochemical prognostic factors (ERα immunoexpression; Ki67 index) to assess the prognostic value of these miRNAs. Although the upregulation of miR-18a and miR-18b has been previously demonstrated in canine mammary carcinomas [31,32], to the best of the authors’ knowledge, no study has previously explored the correlation between these miRNAs and their main target mRNA (i.e., *ESR1* mRNA), as well as the correlation between miR-18 and miR-18b expression, ERα immunoexpression and Ki67 index in CMTs. The results obtained here demonstrate a negative correlation between the expression of miRNAs and their target gene *ESR1* in relation to histological and immunohistochemical prognostic factors. Notably, a significant upregulation of miR-18a and miR-18b was observed in malignant CMTs compared with the normal/hyperplastic mammary gland. Moreover, miR-18a was also overexpressed in malignant CMTs with the benign tumor types. Conversely, the expression of target *ESR1* mRNA was significantly downregulated in malignant tumors compared with both benign CMTs and the normal/hyperplastic mammary gland. It is noteworthy that the expression of miRNAs was negatively correlated with ERα immunoexpression, with a significant overexpression of both miR-18a and miR-18b in CMTs with the lowest ERα score (i.e., 4–6). Moreover, a significant positive correlation was found between the Ki67 index and miRNA expression only in malignant tumors, with the highest miRNA expression level in tumors with the highest proliferative index.

Commonly, miR-18a and miR-18b, and their cluster members, are described as onco-microRNAs, as they show increased expression in more aggressive tumor types [12,31,32]. Especially in canine mammary cancer, miR-18a is an oncomiR found to be upregulated in exosomes isolated from malignant mammary epithelial cell lines [31] and overexpressed in sera from canine patients with metastatic CMTs compared with those with non-metastatic mammary tumors [32], thus suggesting their role as promising prognostic biomarkers.

Based on the target gene analysis, *ESR1* mRNA, known to be associated with a risk for CMTs [42], most likely appears to be the main target gene for both miR-18a and miR-18b (miRDB score 99) [30,31]. In addition, these miRNAs are also involved in other levels of epigenetic regulation and many of their gene targets are related to chromatin remodeling processes [30,31]. Compared with miRNA expression levels, an opposite trend in the expression pattern of the *ESR1* target gene was observed in this study, with a significantly lower expression of *ESR1* mRNA in malignant CMTs compared with both the non-neoplastic mammary gland and benign tumors. Data concerning *ESR1* gene expression in CMTs are controversial [20,27,43,44,45], and although a significantly lower gene expression is commonly reported in carcinomas compared with adenomas and normal mammary glands [20,27,43], some studies found no differences in *ESR1* mRNA expression between normal and neoplastic canine mammary tissues [44,45].

In the dog, the *ESR1* gene encodes for ERα in the mammary gland, and a reduced gene expression observed in malignant tumors here closely reflects reduced ERα immunoexpression. Based on the semi-quantitative scoring system, normal and hyperplastic mammary glands showed higher ERα immunoexpression (i.e., TS = 8), reflecting their higher expression level of *ESR1* mRNA, while the total score for ERα ranged from 4 to 8 for benign CMTs, and from 4 to 7 for malignant tumors, reflecting the decreasing trend in *ESR1* gene expression. In general, *ESR1* gene expression has a similar pattern to ERα immunoexpression, with a loss of gene and receptor expression in high-grade carcinomas [20,21], and lower protein scores in more aggressive carcinomas compared with benign tumors and normal mammary glands [46]. In our study, only two malignant CMTs were considered to be ERα-negative (i.e., intraductal papillary carcinoma, grade I; solid carcinoma, grade III); however, *ESR1* mRNA was also amplified from these ERα-negative tumors. In agreement with our results, *ESR1* mRNA expression was observed in ERα-negative CMTs [27], and variations in mRNA and protein levels were attributed to several mechanisms, including post-transcriptional gene regulation. Indeed, unlike human breast cancer, lower *ESR1* gene expression in more aggressive CMTs does not appear to be regulated by DNA methylation [27] and would be induced by causes that are still unknown.

Changes in hormone receptor expression and activity are crucial in the initiation and progression of mammary tumors, and these changes can be expected to reflect dysregulation of the miRNA targeting steroid receptors [29,47,48]. Indeed, *ESR1* mRNA is an important predicted target for miR-18a and miR-18b, and the relationship with miRNAs dysregulation and canine mammary carcinogenesis has already been discussed [30], while a significant negative correlation between miR-18a and miR-18b dysregulation and a loss of ERα immunoexpression has been observed here.

It is noteworthy that both miRNA expression levels showed a significant negative correlation with ERα immunoexpression, with the highest miRNA expression level in CMTs having the lowest ERα immunoexpression, and although it has long been known that human and canine mammary neoplasms lose ERα expression along with increased stage and grade, our findings may indicate that miR-18a and miR-18b contribute to a loss in hormone receptor activity [24,25]. In agreement, the upregulation of miR-18a and miR-18b has been associated with an ER-negative status, high proliferation rate and poorer prognosis in women with breast cancer [49,50], and these miRNAs have been found to be largely expressed in the intratumoral stroma and in the stroma directly surrounding ER-negative tumors with a large number of tumor-infiltrating lymphocytes (TILs) [51]. Besides a poor disease prognosis and greater malignancy, the downregulation of ERs leads to low responsiveness to endocrine therapy [52], as cancer assumes a more aggressive phenotype with estrogen-independent growth. Therefore, in addition to the prognostic value, the expression of ERα would allow one to predict a response to anti-estrogenic hormone therapy, as patients with ERα-positive tumors may benefit from estrogen ablation or ERα pharmacological blockage [23]. Therefore, a loss in ER expression complicates the selection of breast cancer treatment strategy.

In this study, we found a total ERα score of 6 to 7 in three malignancies (i.e., tubulopapillary carcinoma, grade I; mixed carcinoma, grade II; solid carcinoma, grade III). In breast cancer, the impact of oncogenic miRNAs on estrogen receptor expression and activity largely depends on the context of the disease [10] and noteworthy, in advanced stages, usually characterized by a decrease or loss in ER expression, many oncogenic miRNAs can induce ER re-expression [10]. Thus, the regulation of miR-18a and miR-18b would appear to be one of several epigenetic mechanisms controlling hormone receptor activity and expression in mammary cancer. However, the restoration of ER expression in advanced stages significantly improves the sensitivity of breast cancer to systemic therapy [47]. In contrast, Luengo et al. [48] reported poorer survival of breast cancer patients who received neoadjuvant chemotherapy with miRNA-18a expression in residual tumors. Indeed, miR-18a expression downregulates ER expression and decreases sensitivity to tamoxifen [48], thus suggesting that the suppression of miR-18a expression would be beneficial for patients with breast cancer. ER expression was also confirmed as being a differentiation marker associated with better prognosis in canine patients with mammary cancer, as ERα-positive carcinomas showed significantly lower proliferative activity (Ki67 values) and longer survival compared with ERα-negative tumors [21].

Regarding the tumor proliferation rate, a higher Ki67 index was observed here in malignant CMTs and a significant but weak negative correlation was observed between miR-18a gene expression and CMTs with Ki67 < 33.3%, while a significant positive correlation was observed between miR-18a and miR-18b expression and malignant tumors with a higher Ki67 index than benign CMTs. Based on the Ki67 index, CMTs were also distinguished into high and low proliferative tumors, and there is ample evidence that malignant tumors with increased tumor cell proliferation, as measured by the Ki67 index, are associated with a poor prognosis [53,54]; furthermore, the significant positive correlation between the Ki67 index and miRNA expression observed here confirms the prognostic value of these two biomarkers.

## 5. Conclusions

According to the results found here, the upregulation of miR-18a and miR-18b in canine mammary carcinomas is inversely correlated with the expression of *ESR1* target mRNA and closely reflects the progressive loss of ERα immunoexpression, thus, representing a potential epigenetic mechanism in the downregulation of ERα. Furthermore, the overexpression of miR-18a and miR-18b is observed in CMTs with increased tumor cell proliferation as measured using the Ki67 index. Taken together, these results suggest a central role of miR-18a and miR-18b in the pathophysiology of canine mammary cancer, also representing promising biomarkers with predictive and prognostic value. However, more extensive and detailed investigations in the epigenetic field are needed to better understand the influence of this area on canine mammary tumors. Finally, an epigenetic view of cancer is crucial to improve therapeutic strategies and develop new therapeutic tools in the near future.

## Figures and Tables

**Table 1:** Allred scoring system for ERα immunohistochemical analysis [35].

Percentage of Cell Labeling	Score	Intensity of Labeling	Score
No labeling	0	Absent	0
<1%	1	Weak	1
1–10%	2	Moderate	2
10–33%	3	Strong	3
33–66%	4		
66–100%	5		

**Table 2:** Sequences of the primers used in this study [27,39,40].

Gene	Forward Primer	Reverse Primer	Accession No.
Estrogen receptor 1 (*ESR1*)	AGCTCCTCCTCATCCTCTCC	AGGTCGTAGAGAGGCACCAC	
Ribosomal protein S19 (*RPS19*)	CCTTCCTCAAAAA/GTCTGGG	GTTCTCATCGTAGGGAGCAAG	XM_533657
Hypoxanthine phosphoribosyltransferase 1 (*HPRT*)	TGCTCGAGATGATGAAGG	TCCCCTTGACTGGTCATT	NM_000194

**Table 3:** Histological classification, Allred Score and Ki67 indexes for canine mammary samples. TS = total score. * ER-negative tumors [24,36].

Samples	Histological Classification	*n*	Grade of Malignancy	Lymphatic Invasion	TS	Ki67 Index (%)
Controls (*n* = 4)	Normal Mammary Gland	3			8	-
	Lobular Hyperplasia	1			8	52.8
Benign Tumors (*n* = 7)	Tubular Adenoma	1			8	12
	Tubulopapillary Adenoma	1			8	14.6
	Complex Adenoma	1 1			8 7	32.4 36.8
	Intraductal Papillary Adenoma	1 1			6 4	77.2 16.8
	Benign Mixed Tumor	1			6	19.6
Malignant Tumors (*n* = 15)	Tubular Carcinoma	1 1	I III	Yes	5 5	60 30
	Tubulopapillary Carcinoma	1	I		7	25.5
	Complex Carcinoma	1 1 1	I I I		4 5 5	58.4 54.6 17.2
	Intraductal Papillary Carcinoma	1 1	I I		2 * 5	47 19.2
	Mixed Carcinoma	1 1	I II		4 7	7 75
	Solid Carcinoma	1 1 1 1	II III III III		4 4 6 3 *	54 52.6 61.8 60.2
	Inflammatory Carcinoma	1	III	Yes	4	87.6

**Table 4:** Expression levels of miRNAs in CMTs based on ERα immunoexpression and Ki67 index. Data are presented as mean ± SD. Gene expression levels are intended as fold change normalized to endogenous controls using Formula 2 ΔΔCq.

ER Score	*n*	miR-18a	miR-18b
4–6	14	1.86 ± 0.90	2.03 ± 1.41
7–8	6	1.29 ± 0.57	1.64 ± 0.38
**Ki-67 Index**			
<33.3% (19.39 ± 7.93)	10	1.41 ± 0.78	1.62 ± 0.58
>33.3% (60.43 ± 13.86)	12	1.88 ± 0.82	2.31 ± 1.46

## Data Availability

Not applicable.
